# Further evidence for malaria elimination failutre on the island of Sainte Marie (Madagascar)

**Published:** 2011-01-10

**Authors:** Voahangy Andrianaranjaka, Lanto Alisoa Ranarivelo, Anatole Dadare, Rogelin Raherinjafy, Stephane Rabearimanana, Benjamin Ramarosandratana, Philémon Tafangy, Milijaona Randrianarivelojosia

**Affiliations:** 1 Institut Pasteur de Madagascar, Antananarivo (101), Madagascar; 2 District de Santé Publique, Sainte Marie (515), Madagascar; 3 Ministère de la Santé Publique, Ambohidahy, Antananarivo (101), Madagascar

## 

Since 2006, the new malaria elimination programme has been implemented on Sainte Marie Island – a district of approximately 20,000 inhabitants, on the eastern coast of Madagascar. Key malaria interventions include mass distribution of long lasting insecticide-treated nets (LLINs), intermittent preventive treatment using sulfadoxine-pyrimethamine in pregnant women (IPTp) and ACT for treating uncomplicated malaria cases. Over 20,000 LLINs were distributed in 2006. The implementation of this programme on Sainte Marie is expected to generate useful and usable information to inform malaria elimination strategies in the entire country.

As part of the routine monitoring at the health district level, active detection of malaria was carried out in primary school children in Sainte Marie during the rainy season from January 27 to March 3, 2009. Giemsa stained blood smears were examined for malaria parasite at the Institut Pasteur de Madagascar. Also, blood spots were collected. The presence/absence of *pfdhfr* S108N and S108T mutations in *Plasmodium falciparum* isolates was detected by PCR/RFLP method.

Asymptomatic and consenting 524 children participated in this survey. The mean age was 8.8 ± 2.1 years. The malaria prevalence was 20.2% (95%CI: 16.9 – 23.9%) with a predominance of *P. falciparum* malaria (105/106) and a single case of *P. vivax*. Of the 105 isolates *P. falciparum* isolates, two (1.9%) harboured the S108N mutation at position 108 in pfDHFR.

Our findings demonstrate that in three years following the malaria treatment policy change, mutant *P. falciparum* strains potentially resistant to sulfadoxine-pyrimethamine emerge in Sainte Marie. This is considered as an alarming situation given the importance of the IPTp to control malaria in pregnancy. Also, the prevalence of malaria among children above five is still high. This is a further proof of the malaria elimination failure. Overall, this situation resulted from weaknesses in malaria control measures. A single massive distribution round of LLINs in 2006 is not enough. Besides, cyclones hit Sainte Marie every year and most of the nets disappeared with broken houses. We believe that key approaches to achieve malaria elimination in Madagascar are (i) coupling malaria surveillance with interventions with additive innovative approaches such as treating asymptomatic malaria cases with ACT and (ii) on-going maintenance of malaria prevention including the unfailing renewal of LLINs coverage and in door spraying of insecticide.

